# Considerations and Challenges of Resorbable Intramedullary Nailing in Pediatric Forearm Fractures

**DOI:** 10.3390/children12050606

**Published:** 2025-05-06

**Authors:** Gergő Józsa, Tamás Kassai, Marcell Varga, Ádám L. Dávid, Zoltán Tóth, Tibor Molnár, Eszter Antal, Anna Gabriella Lamberti, Hermann Nudelman, Aba Lőrincz

**Affiliations:** 1 Division of Surgery, Traumatology, Urology and Otorhinolaryngology, Department of Pediatrics, Clinical Complex, University of Pécs, 7 József Attila Street, 7623 Pécs, Hungary; toth.zoltan3@pte.hu (Z.T.); molnar.tibor@pte.hu (T.M.); lamberti.anna@pte.hu (A.G.L.); 2 Department of Thermophysiology, Institute for Translational Medicine, Medical School, University of Pécs, 12 Szigeti Street, 7624 Pécs, Hungary; daaxaao.pte@pte.hu (Á.L.D.); antal.eszter1@edu.pte.hu (E.A.); nuhwaao.pte@tr.pte.hu (H.N.); aba.lorincz@gmail.com (A.L.); 3 Department of Pediatric Traumatology, Péterfy Hospital, Manninger Jenő National Trauma Center, 17 Fiumei Street, 1081 Budapest, Hungary; kassai.tamas@obsi.hu (T.K.); drvmarcell@gmail.com (M.V.)

**Keywords:** pediatric, diaphyseal, forearm, fracture, complication, resorbable, PLGA, intramedullary

## Abstract

**Background**: Pediatric diaphyseal forearm fractures, often caused by sports or leisure activities, require treatment based on fracture stability. While stable fractures can be managed conservatively, unstable fractures typically require surgery, with elastic stable intramedullary (IM) nailing (ESIN) being the gold standard. Bioabsorbable IM nails (BINs) offer an alternative by eliminating the need for implant removal surgery. **Methods**: Between May 2023 and January 2025, we consecutively managed 161 children with poly-L-lactic-co-glycolic acid (PLGA) BINs in two healthcare centers for diaphyseal forearm fracture and evaluated every reported difficulty and complication of resorbable IM nails. **Results**: Seven unique peri- or postoperative events occurred during the study period. Even with a high success rate, some complications occurred during the study period. Difficulties and complications were mainly dependent on the surgical technique. Iatrogenic complications such as bone cortex perforation and implant end split were evaluated, along with anatomical variations like focal and general medullary cavity stenosis narrowings that affected implant insertion. Secondary malalignment, one early (2 months) refracture, and one recurrent fracture (2 years) were also noted. **Conclusions**: Although BINs reduce the need for a second surgery, careful planning, technique, and follow-up are crucial for optimal outcomes. Further research is needed to assess long-term results and complications.

## 1. Introduction

Fractures are decidedly common injuries in the pediatric population, accounting for approximately 25% of all childhood injuries, with radial and ulnar fractures having the highest incidence, making up 36% of all childhood fractures [1]. The mechanism of injury is mainly accidental trauma resulting from sports or leisure activities [2].

Diaphyseal forearm fractures in children are classified via the Pediatric Comprehensive Classification of Long-Bone Fractures (PCCF) created by the Swiss Arbeitsgemeinschaft für Osteosynthesefragen (AO) Foundation [3]. Treatment methods are determined by the stability and complexity of the fracture morphology. Stable, nondisplaced diaphyseal fractures can be treated conservatively with immobilization utilizing a long arm cast [4,5]. Noonan and Price described the conservative treatment limits of pediatric forearm shaft fractures by considering patient age, fracture angulation, rotation around the longitudinal axis of the affected bone, and bayonet apposition [6]. Intrinsically unstable fractures require operative treatment. Oblique, spiral, multifragmentary, completely displaced, converging fractures, and both-bone forearm fractures at the same level are some examples of unstable fracture morphology.

Elastic stable intramedullary (IM) nailing (ESIN) is considered the gold standard operative treatment method for unstable diaphyseal forearm fractures [7,8]. ESIN is a minimally invasive procedure that avoids a large incision at the fracture site, thereby significantly reducing the risk of infections, bleeding, and hypertrophic scarring, in contrast to an open approach using plate and screw fixation. ESIN also leads to minor soft tissue damage upon subsequent implant removal. In recent years, however, several articles have discussed primary surgical complications, which may occur after implantation, during the procedure, or after nail removal [9,10,11,12,13,14,15,16]. Complication rates range from 8.76% [17] to 50%, with up to 80% of multiple symptoms [11] and varied outcomes reported in different studies [18,19]. It may result in transient nerve palsy, malunion, or postoperative compartment syndrome [10]. Synostosis, muscle entrapment, osteomyelitis, hardware migration or malplacement, loss of reduction, and significant range of motion reduction have also been described [11,12]. Depending on the entry point, during a dorsal approach, rupture of the extensor pollicis longus (EPL) tendon occurred relatively frequently [13,20]. Meanwhile, the metal end may cause skin irritation and skin perforation, regardless of the entry point [12,20]. Post-operative radial bow was significantly higher in the ESIN group, without affecting forearm movement [21]. Several authors have also reported complications related to metal removal, such as injury to the sensory branch of the radial nerve [14,15]. Younger children seem to have a higher refracture rate [22].

In recent years bioabsorbable IM nails (BINs) have been developed and used in the treatment of pediatric diaphyseal forearm fractures. They aim to eliminate the need for a second surgery for metal removal and reduce the total required anesthesia, exposure to radiation through imaging, length of hospital stay (LOS), discomfort by the procedures, and therefore the whole financial burden of an additional intervention [23]. BINs require only one minimally invasive surgical procedure for insertion and thereafter dissolve within the medullary canal at a rate slower than bone healing [24].

Our first descriptive publications reporting the use of absorbable implants in pediatric forearm fractures provided a thorough presentation of the surgical technique and the advantages and disadvantages of the method [23,25]. However, there is very little data in the international literature regarding the technical difficulties and complications related to resorbable IM implants, which differ from traditional titanium elastic nailing in many aspects [26].

Therefore, this study aims to describe the intraoperative difficulties and potential complications after poly-L-lactic-co-glycolic acid (PLGA) BIN management of pediatric diaphyseal fractures.

## 2. Patients and Methods

### 2.1. Study Design and Patient Selection

The Department of Pediatrics at the University of Pécs, Clinical Complex and the Department of Pediatric Traumatology, Péterfy Hospital, Manninger Jenő National Trauma Center are involved in an ongoing prospective multicenter clinical study analyzing the treatment of pediatric diaphyseal forearm fractures with the IM PLGA implants (Activa IM-Nail™, Bioretec Ltd., Tampere, Finland). This study aims to observe and document any intraoperative difficulties and postoperative complications with PLGA BIN treatment between May 2023 and January 2025.

Out of 161 children, seven patients met the inclusion criteria, which were (1) pediatric patients (≤18 years old), (2) suffering a diaphyseal forearm fracture, and (3) receiving PLGA IM implants. No patients were excluded from the study. However, the contraindications of PLGA BINs would have restricted its application in the cases of (1) bilateral- (2) oblique spiral-, (3) multi-fragmentary-, or (4) epiphyseal fractures, (5) local infection, (6) poor compliance, and (7) bone re-modelization affecting comorbidity or (8) medication; however, no patient was admitted with any of these circumstances during the investigation period.

### 2.2. Intervention

Our IM implant was a PLGA-based internal fixation, that supported the stabilization of the fracture and bone healing. Once placed, the polymer undergoes hydrolytic breakdown, releasing degradation products that feed into the citric acid cycle, where they are converted to water and carbon dioxide (CO_2_) [27]. The local drop in pH created by this process helps pace the gradual disappearance of the BIN. By around 9–12 months, decomposition is typically finished—aligning with the timeframe needed for fracture repair in children and erasing the necessity for a second operation to remove fixation material [28,29].

To aid surgeons in confirming proper placement, β-tricalcium phosphate (β-TCP) markers are included in the implant, making it visible under fluoroscopy. Varying the ratio of lactic to glycolic acid in the PLGA allows control over how quickly the nail erodes and how it performs structurally. These adjustments support fracture alignment while offering a biodegradable solution that meets the demands of a growing skeletal system.

### 2.3. Operative Algorithm

Minimally invasive implantation of PLGA nails follows several essential steps, trying to balance necessary immobilization for fracture healing and the eventual return to normal activities. After standard skin cleansing, disinfecting, and induction of general anesthesia, the child is positioned supine with the affected forearm on a radiolucent support. Perioperative comfort is ensured by a tailored analgesic protocol involving 0.1–0.2 mg/kg nalbuphine (Nubain, ALTAMEDICS GmbH, Cologne, Germany) and, if warranted, sedation with midazolam (Dormicum, Egis Gyógyszergyár Zrt, Budapest, Hungary), following parental agreement.

Small radial incisions over the distal radius and lateral incisions over the proximal ulna provide entry to the cortical bone using an awl or drill. For the radial bone, the drill is guided perpendicular to the cortex initially, then reoriented to a more acute angle in line with the diaphysis. Sequentially, the medullary canals are expanded with dilators matching the implants’ diameters (2.7 or 3.2 mm) and lengths (200, 300, or 400 mm). Selecting an appropriately sized implant is vital; it should fit comfortably within the narrowest portion of the intramedullary space.

Once canals are prepared, the PLGA nails are introduced with an inserter, avoiding rotational stresses that might impair fracture alignment. Fluoroscopic checks confirm correct positioning, aided by the β-TCP tips. Any excess nail protruding beyond the cortex is trimmed, ensuring smooth bone-implant junctions that minimize soft-tissue discomfort. Each small incision is then joined with absorbable material, using intracutaneous suture lines in the skin.

Post-surgical care usually involves placing the arm in an above-elbow cast with the elbow flexed at 90 degrees for approximately 4–6 weeks, allowing primary bone stability to consolidate. Strenuous activity, including sports, is discouraged for 4–6 months to prevent re-injury. Operative protocols are compared between PLGA and titanium nailing in Table 1.

## 3. Results

### 3.1. Case 1: Iatrogenic Cortex Perforation

**Figure 1:**
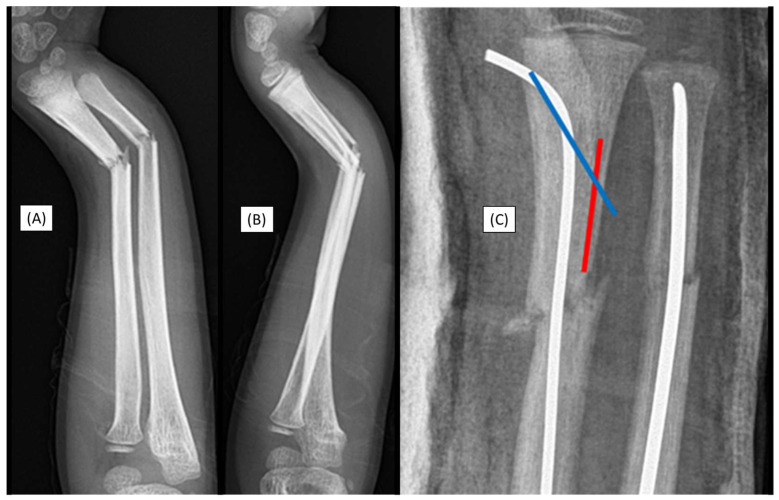
Primary X-rays of a distal diaphyseal forearm fracture from (**A**) anteroposterior (AP) and (**B**) lateral views. After the insertion of the dilator (blue line), the opposite cortical was perforated (red line). Following this intraoperative complication, elastic nails were inserted, and both bones were stabilized with ESINs. Postoperative X-ray demonstrates good alignment (**C**).

During a handball match, the left forearm of a 12-year-old boy was injured. X-rays confirmed a distal dia-metaphyseal forearm fracture (Figure 1A,B). Preparation of the medullary cavity is crucial when using absorbable IM implants. Following the closed reduction of a fracture, due to instability, the (2.7 mm) dilator was introduced through a typical radial approach, which resulted in cortical perforation on the opposite side (Figure 1C).

Although this case did not involve an implant-related complication, it directly relates to the challenges associated with the surgical procedure. Therefore, when such intraoperative complications are observed, the use of absorbable implants is not recommended.

### 3.2. Case 2: Narrow Medullary Cavity

**Figure 2:**
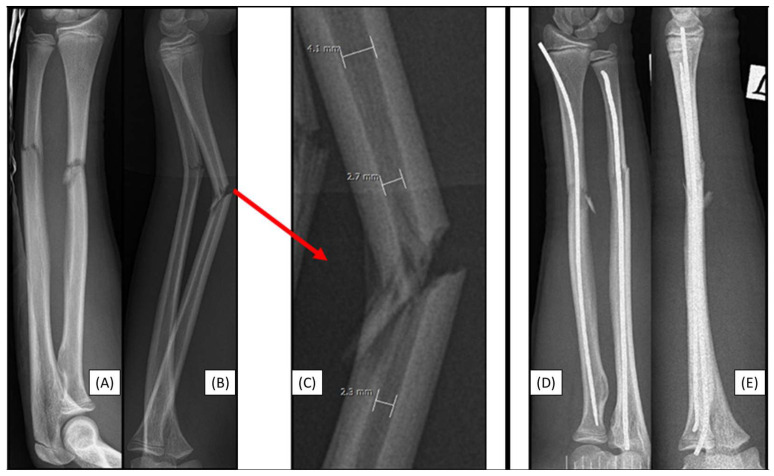
Preoperative X-rays from AP (**A**) and lateral (**B**) aspects of a left-sided both-bone diaphyseal forearm fracture. A red arrow marks the origin of the magnified image (**C**) showing the measurement of the medullary canal both distal and proximal to the fracture. Both bones were stabilized with ESINs, visible from AP (**D**) and lateral (**E**) views.

A 14-year-old boy was scheduled for surgical treatment due to a displaced, unstable middle-third forearm fracture (AO type: 22-D/2.1.) on the left side. As with any surgery, preoperative planning is crucial, including measuring the diameter and shape of the medullary cavity. Preoperative measurements indicated that the medullary cavity diameter was narrow. Despite multiple attempts, the introduction of the 2.7 mm dilator failed during the preparation of the radius medullary cavity. As we were unable to prepare the medullary cavity, the use of absorbable IM implants was unfeasible (Figure 2A–C). Radius stabilization was therefore performed with a two mm ESIN, while the ulna was steadied with a 2.5 mm nail (Figure 2D,E). Again, while this case was not a direct, implant-related complication, it illustrates a technical challenge connected to the procedure.

### 3.3. Case 3: Apical Implant Splitting

**Figure 3:**
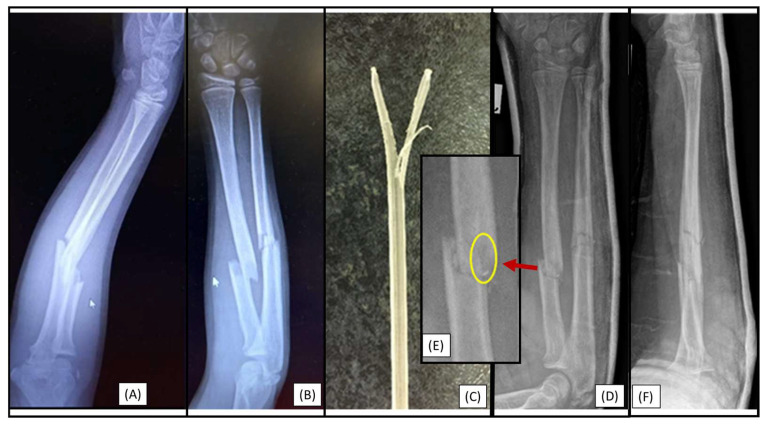
Initial X-rays of the diaphyseal forearm fracture (lateral (**A**), AP (**B**)) and the split end of the resorbable IM nail (**C**). Postoperative images (lateral (**D**), AP (**F**)) show good alignment of the bones. However, the red arrow highlights an area from Figure 3D, where the magnified AP view (**E**) demonstrates a two mm displacement of the radius, which caused the injury of the implant. A yellow circle shows the β-TCP tip and location where it left the medullary cavity.

Trampoline-fall injury of a 10-year-old child resulted in a complete (22-D/4.1.) fracture of the right forearm. Following the closed reduction of a complete proximal third forearm fracture and proper preparation of the medullary cavity, the introduction of the (3.2 mm) absorbable IM PLGA nails at the fracture site was difficult (Figure 3A,B). BINs could not be passed through the fracture gap, and the β-TCP marking was visible outside the bone’s medullary cavity, projecting onto the interosseous membrane. After removal of the implant, we observed that the end of the nail had split (Figure 3C). Implant failure can be explained by challenges in surgical technique; after losing the precise reduction, the introduction of the BIN was forced, causing it to break. Therefore, gentle implant insertion in case of obstruction is preferred, like withdrawing and repeating the preparation of the medullary cavity with an appropriately sized dilator.

### 3.4. Case 4: Focal Medullary Cavity Stenosis

**Figure 4:**
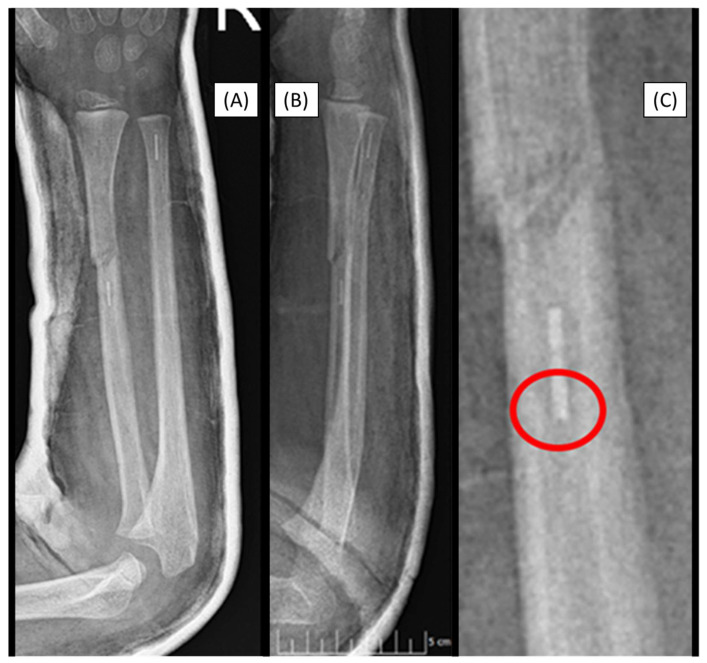
Postoperative X-rays (AP (**A**), lateral (**B**)) depict the fracture stabilization in good alignment. The β-TCP marker is visible in the right radius at the level of the diaphysis, two cm proximal to the fracture site. The cause is visible in the magnified image (**C**), where the medullary cavity narrows, creating a sandglass effect, marked by the red circle.

After a closed reduction of a right diaphyseal forearm fracture (22-D/2.1.), the medullary cavity was prepared in an 8-year-old child. However, the resorbable (2.7 mm) IM implant became stuck when passing through the fracture gap. Medullary cavity shape was the etiology behind the blockage, which could not be adequately prepared even with the dilator. Consequently, the implant became stuck, though, we did not attempt to force its removal (Figure 4).

### 3.5. Case 5: Secondary Malalignment

**Figure 5:**
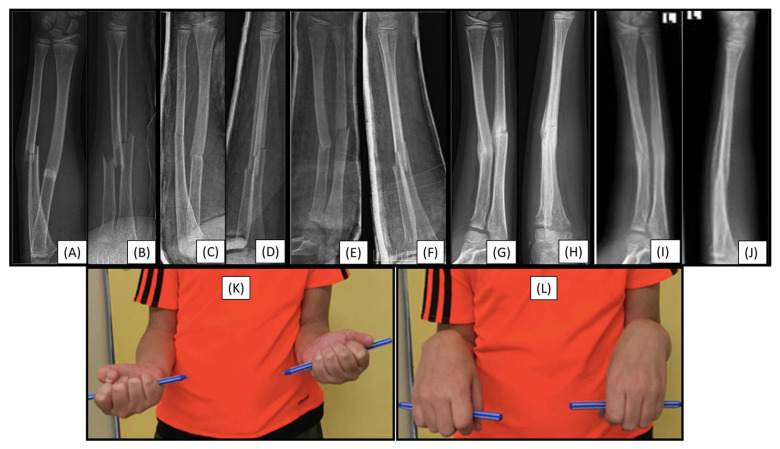
Preoperative (from AP (**A**) and lateral (**B**) views), and immediately postoperative (AP (**C**), lateral (**D**)), control radiographs. Follow-up X-rays were taken one week (AP (**E**), lateral (**F**)), eight weeks (AP (**G**), lateral (**H**)), and six months postoperatively (AP (**I**), lateral (**J**)), exhibiting good callus formation and remodeling. Left wrist supination showed a 10° deficit (**K**), with no discrepancy in pronation range (**L**).

After a 12-year-old patient who had sustained a both-bone proximal diaphyseal forearm fracture (22-D/4.1.) in the left arm (Figure 5A,B), secondary malalignment was observed. Postoperative visualization initially showed adequate alignment subsequent to reduction and stabilization with PLGA BINs (Figure 5C,D).

Control X-rays performed one week postoperatively showed a mild secondary malalignment, with shaft axis angulation of <15° (Figure 5E,F). On an imaging follow-up, eight weeks after surgery, the angulation persisted, and a callus had formed at the fracture sites (Figure 5G,H). Clinically, the patient showed a 10° deficit in supination of the left wrist (Figure 5K), without pronation insufficiency (Figure 5L). Follow-up of the child is still ongoing.

### 3.6. Case 6: Early Refracture

**Figure 6:**
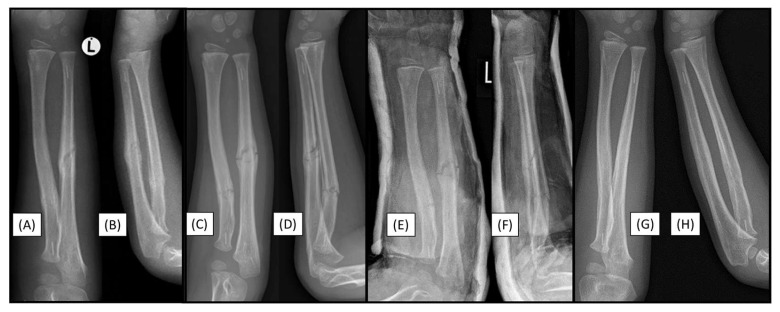
Control X-rays four weeks postoperatively showed good alignment from AP (**A**) and lateral (**B**) aspects. Radiographs at re-presentation eight weeks postoperatively, indicated refracture (AP (**C**), lateral (**D**)). Control X-rays after cast application upon re-presentation with refracture, showing good alignment (AP (**E**), lateral (**F**)). Follow-up radiographs taken six months postoperatively indicate adequate fracture healing (AP (**G**), lateral (**H**)).

Closed reduction and immobilization in a long-arm plaster cast were the initial management of a 6-year-old boy left both-bone diaphyseal forearm fracture (22-D/2.1.). However, a control X-ray performed five days after cast application revealed increased angulation of the radius despite conservative treatment, indicating operative intervention was needed. Subsequently, the fractures were treated with closed reduction and resorbable IM nailing. Plaster cast removal was four weeks postoperatively, and the control X-ray showed satisfactory alignment (Figure 6A,B).

Eight weeks after the operation, the patient experienced an accident, falling while jumping on a trampoline. Despite our recommendation to avoid returning to sports for 4–6 months, this case, although not directly implant-related complication, underscores the importance of adhering to post-operative guidelines. Upon visualization, an X-ray of the left arm revealed a refracture with mild angulation (Figure 6C,D). Therefore, a long-arm cast was applied, and a control X-ray taken post-procedure showed appropriate alignment (Figure 6E,F). Six-month follow-up radiographic images demonstrated good callus formation and maintained alignment (Figure 6G,H); moreover, the clinical examination confirmed a full range of motion (ROM).

### 3.7. Case 7: Recurrent Fracture

**Figure 7:**
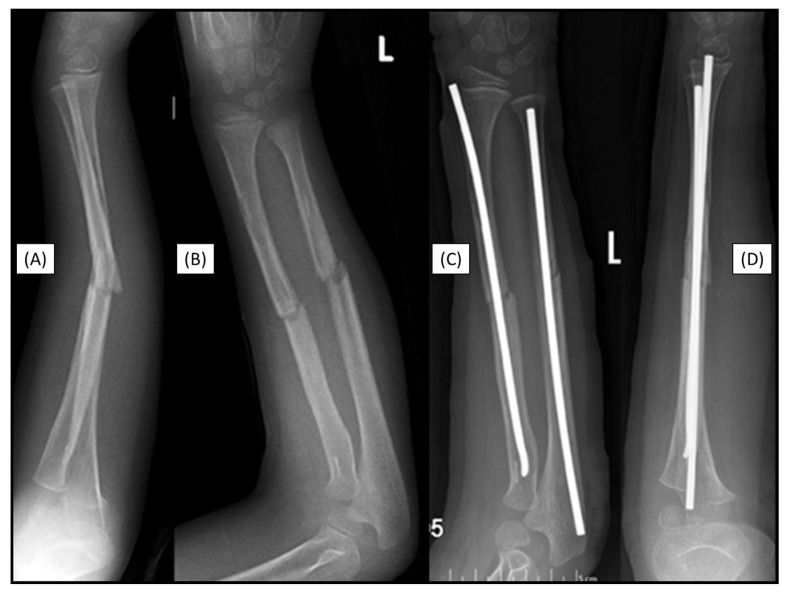
Markers are visible in both bones two years after the primary treatment with BINs (lateral (**A**), AP (**B**)). Postoperative X-rays show the perfect alignment of the forearm, stabilized with ESINs (AP (**C**), lateral (**D**)).

Left diaphyseal forearm fractures (22-D/2.1.) of a 7-year-old boy were primarily fixed with absorbable PLGA IM nails. Two years after the closed reduction and BIN fixation of the fractures, a repeated, non-implant-linked forearm diaphysis fracture occurred after a fall from a horse (Figure 7A,B). Following closed reduction, the bones were stabilized again, now with titanium ESINs. During the surgery, no resistance was encountered in the IM space, and the medullary cavity was free, allowing for smooth insertion of the titanium implant without difficulties (Figure 7C,D).

## 4. Discussion

Pediatric diaphyseal forearm fractures are common injuries in children, where the treatment approach depends on the stability and morphology of the injury. Unstable (such as those involving significant displacement), rotational, or both-bone fractures, typically require surgical intervention [4]. Currently, titanium ESIN is considered the gold standard for treating unstable pediatric forearm fractures, despite its well-documented drawbacks [8,11,12]. Utilizing ESIN, a trial by Kruppa et al. noted 18 complications out of 202 children (8.91%); ten (4.95%) refractures, three EPL ruptures (1.49%), 2–2 malunions and limited ROMs (0.99–0.99%), and an infection (0.50%), however, they did not report nerve damage, skin irritation or perforation rates [19]. Additionally, the research mentioned 18 complications in 10 out of 20 children (50%) including hardware migration and muscle entrapment when using traditional IM nailing [11]. Another trial discussed six (5.88%) refractures in 102 pediatric patients with forearm fractures, along with seven (6.86%) radial neuropraxia and five (4.90%) superficial wound infections with a 17.65% overall risk. Internal fixation with steel plates and screws can be performed as an alternative for titanium IM nails, preferably in skeletally mature or near-mature children [31]. Delayed unions and nonunions are rare but seem to be slightly more common with ESIN compared to internal fixation, although with a statistically insignificant difference [32]. Accompanying extensive soft tissue damage, compartment syndrome, open, and complicated multi-fragmentary fracture morphologies may be treated with fixateur externe [31].

Recent developments have introduced softer, resorbable IM nails as a more physiological alternative to titanium hardware. PLGA implants are capable of eliminating the need for implant removal surgery, reducing the risk of complications associated with a second operation. Perhomaa et al. found that 15/26 (57.69%) BINs completely dissolved, and the rest were almost entirely (42.31%) resorbed within the bone over four years, although β-TCP-tips were still visible in 92.31% of cases [26]. BINs helped in avoiding a second anesthesia, imaging-related ionization, emotional distress, and the financial burden of an additional procedure [23,26,33]. Furthermore, in our preceding diaphyseal fracture PLGA management study, we investigated Vancouver Scar Scale (VSS) scores and satisfaction rates which were found excellent, though significantly differing (*p* = 0.020) when rated by medical professionals (mean VSS: 0.55) or guardians (1.13) [20]. ROMs showed negligible differences compared to the unoperated side one-year post-surgery. Nevertheless, despite these promising benefits, employing bioabsorbable implants is not without challenges.

In a prior study, directly comparing PLGA BINs to ESIN, 7.32% (3 out of 41 children) and 24.44% (11 out of 45 patients) overall complication rates were noted, respectively [33]. Among them, two refractures (4.88%) and one implant-end split (2.44%) occurred with PLGA, and five (11.11%) skin irritations, two (2.22%) skin perforations, three (3.67%) radial nerve injuries and one refracture (2.22%) with ESIN. In this series, we further investigated seven difficulties out of 161 scenarios (4.35% of all cases) associated with the usage of BINs, in the treatment of pediatric forearm fractures.

For instance, inadequate implant introduction resulted in cortical perforation, a complication also reported with traditional elastic nailing [34]. Despite biomechanical studies suggesting high PLGA flexibility (elastic modulus: 2–4 GPa), forced insertion occasionally caused apical implant splitting, due to their lower ultimate tensile (~120 MPa) and compressive strengths (~100 MPa), and fracture toughness (~2 MPa·√m) [35,36] compared to titanium alloys (~110 GPa, ~950 MPa, ~900 MPa, ~95 MPa·√m, respectively [37]). However, BINs more closely match the characteristics of a young cortical bone (~12 GPa, ~90 MPa, ~140 MPa, ~4 MPa·√m, respectively [38]), therefore, they may reduce stress shielding and its consequences, such as osteopenia and implant loosening. Furthermore, flexible polymer fixation permits controlled micro-motion, which can enhance secondary healing (callus formation), and better accommodate the rapid healing and remodeling capacity of children. PLGA screws were already successfully utilized in pediatric ankle fractures, therefore their mechanical attributes are sufficient for load-bearing, though precise weight limits are still investigated for minimizing risks [39]. Several publications address the morphology of the pediatric medullary cavity, which can influence the choice of treatment method [4,40]. In two cases, the medullary cavity exhibited either a sandglass-shaped narrowing or a diffuse constriction, preventing smooth insertion. Preoperative imaging and planning, performing accurate measurements of cavity morphology, and utilizing appropriate-sized dilators with a cautious surgical technique are therefore essential for BIN treatment. Postoperative complications included one case of secondary malalignment, which resolved successfully with adequate callus formation and without functional impairment. Two additional refractures occurred following novel high-energy traumas—one from a trampoline and another from a bicycle accident. Thus, careful patient selection and thorough postoperative follow-up are necessary to promptly identify and manage potential complications, ensuring optimal healing outcomes.

Limitations of this trial include its non-comparative nature, small population, lack of randomization, and no blinding. Advanced imaging could have revealed further aspects, like resorption rates and precise morphology [26]. Objective techniques have been developed to evaluate scar tissue by measuring blood flow, color, and elasticity, however, due to their relatively high price and the generally excellent cosmetic results after minimally invasive procedures, these tests were not performed [41]. Visual analog- (VAS) and Face, Legs, Activity, Cry, Consolability (FLACC) scale usage could have been employed to quantify pain levels.

## 5. Conclusions

Titanium alloys provide approximately an order of magnitude superior absolute mechanical performance compared to PLGA; however, tunable BIN degradation enables transient stabilization. While the increased utilization of resorbable nails in pediatric forearm fractures offers potential benefits (reduced anesthesia and radiation dosage, child distress, LOS, and costs), it is not without its challenges.

Medullary cavity anatomy variability and the potential for surgical and other complications such as implant failure, cortical perforation, and refracture must be carefully considered when choosing the appropriate treatment method. Further, randomized-controlled studies are needed to better understand the long-term outcomes, weight-bearing limits, and complications associated with PLGA implants, as well as to refine the surgical techniques required to minimize complications in pediatric fractures.

## Figures and Tables

**Table 1:** Comparison of PLGA and ESIN surgical techniques.

Surgical Step	PLGA Nails	ESIN [17]
Cortical Access	Gentle drilling: initial perpendicular entry, angled along diaphysis	Simpler insertion
Canal Preparation	Precise sequential dilation, exactly matched to implant size	Less precise sizing needed
Implant Insertion	Careful, rotation-free insertion to prevent splitting	Rotational adjustments allowed; less implant failure risk
Implant Trimming	Trimmed flush to the cortical surface	Often bent externally; may require removal due to irritation
Immobilization	Strict immobilization; casting for 4–6 weeks	Casting may be omitted; active use of the arm is encouraged as tolerated
Return to Sports	Cautious activity restrictions based on severity (4–6 months)	Greater activity tolerance (2–6-month sports restriction) [30]
Hardware Removal	Unnecessary, due to almost complete resorption after 4 years [26]	Second surgery 5–8 months postoperatively, with 2–4-week activity limitation

## Data Availability

Data are contained within the article.
